# Potency of human hematopoietic cells from a novel CD34+ isolation technique

**DOI:** 10.1093/stcltm/szaf067

**Published:** 2025-12-10

**Authors:** James Ropa, Jimin Park, Jessica Newton, So Jeong Kim, Yangshin Park, Jonathan Messer, Justin Blacher, Shabnam Namin

**Affiliations:** Department of Medical and Molecular Genetics, Indiana University School of Medicine, Indianapolis, IN 46202, United States; Department of Medical and Molecular Genetics, Indiana University School of Medicine, Indianapolis, IN 46202, United States; Department of Medical and Molecular Genetics, Indiana University School of Medicine, Indianapolis, IN 46202, United States; Department of Medical and Molecular Genetics, Indiana University School of Medicine, Indianapolis, IN 46202, United States; Department of Biochemistry and Molecular Biology, Indiana University School of Medicine, Indianapolis, IN 46202, United States; 42Bio Inc., Gainesville, FL 32601, United States; 42Bio Inc., Gainesville, FL 32601, United States; 42Bio Inc., Gainesville, FL 32601, United States

**Keywords:** CD34+, cell isolation, hematopoietic stem and progenitor cells, potency, mouse models

## Abstract

Hematopoietic stem and progenitor cells are responsible for maintenance of the immune system and can be a source of cells for therapies. A critical step in studying or utilizing hematopoietic cells is subpopulation isolation. FerroBio is an emerging technology that uses a streamlined, semi-automated approach to isolate CD34+ cells, which are highly enriched for hematopoietic stem and progenitors. This technology also results in isolation of bead-free CD34+ cell samples, in contrast to traditional kits where beads persist following isolation. Here, we showed a side-by-side comparison of FerroBio isolated cells with CD34+ cells isolated by traditional column-based kits. We showed that FerroBio yields similar numbers of CD34+ cells with similar viability, yield, and gated purity and higher overall purity compared to control kits. FerroBio isolated similar numbers of progenitor cells but significantly higher stem cells. *Ex vivo*, cells isolated by FerroBio showed the same ability to form colonies in culture, but FerroBio colony-forming units expanded to a greater extent in liquid culture compared to control. Critically, FerroBio isolated cells had equivalent long-term engraftment capacity with significantly better intermediate-term engraftment compared to control in mouse models of transplantation. Based on microscopy images showing altered morphology co-localized with beads, we inferred that the persistence of magnetic microbeads may be associated with the observed differences. These data demonstrated that specific subpopulations of progenitors from FerroBio isolated CD34+ cells have better potency compared to cells isolated with column-based kits. Thus, FerroBio is a viable strategy for isolating CD34+ cells for research and potentially translational utility.

## Graphical abstract

**Graphical Abstract:**
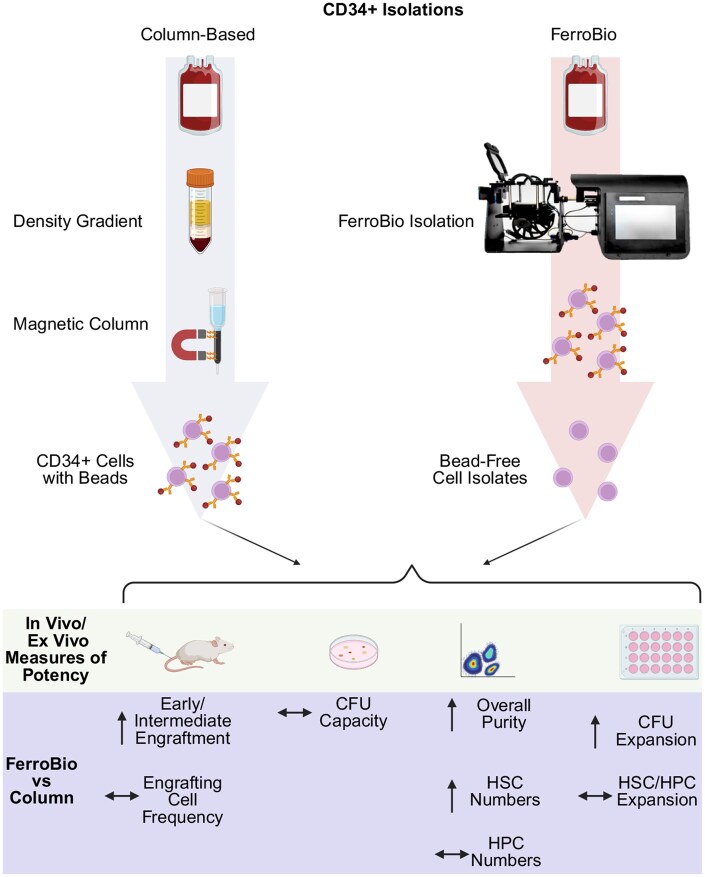

Significance Statement The isolation of CD34+ cells is a critical step in studying hematopoiesis or using these cells for therapeutic purposes. Here, we show that FerroBio™, a novel, semi-automated isolation technology, produces CD34+ cells with some improved potency and fitness metrics compared to standard column-based kits. Thus, this technology may be a technically easier and less damaging method to isolate hematopoietic stem and progenitor cells for research or clinical utility.

## Introduction

Hematopoietic stem (HSCs) and progenitor cells (HPCs) drive the continuous replenishment of the human blood and immune systems.^1^^,^^2^ HSCs/HPCs play critical roles in responses to acute insult or chronic disease and are themselves a source of treatment in the form of hematopoietic cell therapies.^3^^,^^4^ CD34+ cells are a hematopoietic population containing HSCs and HPCs.^5^ Isolated CD34+ cells are frequently used to study healthy and diseased hematopoiesis *ex vivo* and for *in vivo* models of transplantation.^6^ Additionally, CD34+ cells can be used to generate humanized immune systems in mice and other model organisms.^7^ Finally, CD34+ cells are themselves a form of treatment for hematologic disorders in the form of hematopoietic cell therapies such as hematopoietic cell transplantation.^3^^,^^8^ Thus, there is strong interest in isolating CD34+ cells in a manner that best preserves their health and stem or progenitor cell identity.

Immunomagnetic enrichment of CD34+ hematopoietic stem/progenitor cells from bone marrow, mobilized peripheral blood, or umbilical cord blood is an important step in isolating CD34+ cells for further use.^9^^,^^10^ Most existing CD34+ cell isolation methods include a two-step process consisting of (1) depletion of red blood cells and large granulocytes using density gradient centrifugation followed by (2) enrichment of CD34+ cells using immunomagnetic beads targeting the CD34 cell surface antigen.^10^^,^^11^ These methods utilize column or tube-based approaches and typically result in isolation of CD34+ cells with beads still attached to the cells. These commercially available immunomagnetic cell isolation kits are commonly used to obtain enriched isolates of CD34+ cells in laboratory, manufacturing, and clinical settings. However, these methods require significant technical skill and time resources and may impact cell fitness for further utility. This may be due in part to the preprocessing density steps that utilize high speed and long centrifugation steps to reduce blood volume and eliminate red blood cells and platelets.

FerroBio™ is an emerging, immunomagnetic separation technology that has been developed for the isolation of cells and other targets from complex biological fluids. The application of FerroBio™ in this paper is specific to the isolation of research grade CD34+ hematopoietic cells from human umbilical cord blood units (CBUs). Unlike current isolation methods like column-based isolation kits, the FerroBio™ system does not require preprocessing using density gradient centrifugation. Instead, magnetic beads are incubated directly in the cord blood collection bag. Additionally, the cell isolation steps are performed within a patent-pending cartridge, which is not restricted by cell concentration or volume and allows the cells to be magnetically separated without direct contact with a paramagnetic matrix. In contrast, column-based methods are limited by maximum numbers of total and target cells per isolation column, and bead bound cells must eventually be eluted by mechanical force from the matrix to which they bind. FerroBio™ an essential bead removal step after the isolation procedure results in a bead-free CD34+ cell suspension that may then be cryopreserved or used directly for downstream research protocols. This bead removal contrasts with existing technologies including column-based kits where the nanoscale beads remain present following cell isolation.^9^^,^^12^

In this study, we compared FerroBio™ with a commercially available column-based isolation method. We discussed the differences in workflow. We also compared the *ex vivo* and *in vivo* potency of CD34+ cells isolated with FerroBio™ compared to the commonly used column-based isolation method. For these studies, cord blood was split evenly by volume and cells from each half were isolated with either FerroBio™ or control techniques, allowing for direct comparison of the isolation techniques with matched biological replication. Finally, we discussed our findings that cells isolated using FerroBio™ differ from those using common isolation procedures and propose causes that may contribute to these differences.

## Materials and methods

### Cord blood acquisition and pre-cryopreservation analysis

Donated cord blood units (CBUs; *n* = 3 U) for research use were acquired from two independent blood banks, one in north central Florida and the other in Ohio. Incoming CBU attributes were established by the blood banks prior to CD34 isolation (see Supplementary Methods).

### Split bag approach

To eliminate donor variability, cord blood was evenly split by volume. Briefly, blood was removed via syringe through a needleless injection site attached to the collection bag. Blood was then aliquoted into 50 mL conical tubes for the column-based control isolation, and the remaining volume was left in the CBU collection bag for the FerroBio™ isolation. After splitting, each isolation technique was performed concurrently to eliminate the potential influence of storage temperature and blood ageing.

### Column-based control isolation

Blood was preprocessed as previously described.^11^ Briefly, blood was aliquoted, diluted with calcium- and magnesium-free phosphate buffered saline (PBS), and centrifuged to reduce blood volume. Cells were then collected and separated using Ficoll-Paque PLUS density gradient medium (Cytiva, Fisher Scientific). Low density cells from each aliquot were collected, pooled, washed, and resuspended in PBS. Cells were then counted using hemocytometry and labeled with beads using the Miltenyi Biotec CD34 Microbead Kit UltraPure, human (130-100-453), per manufacturer’s instructions. LS columns (Miltenyi Biotec, 130-042-401) were used to separate bead-labeled cells.

### Detailed methods for FerroBio™ isolation

#### Bead binding

**Figure 1.**
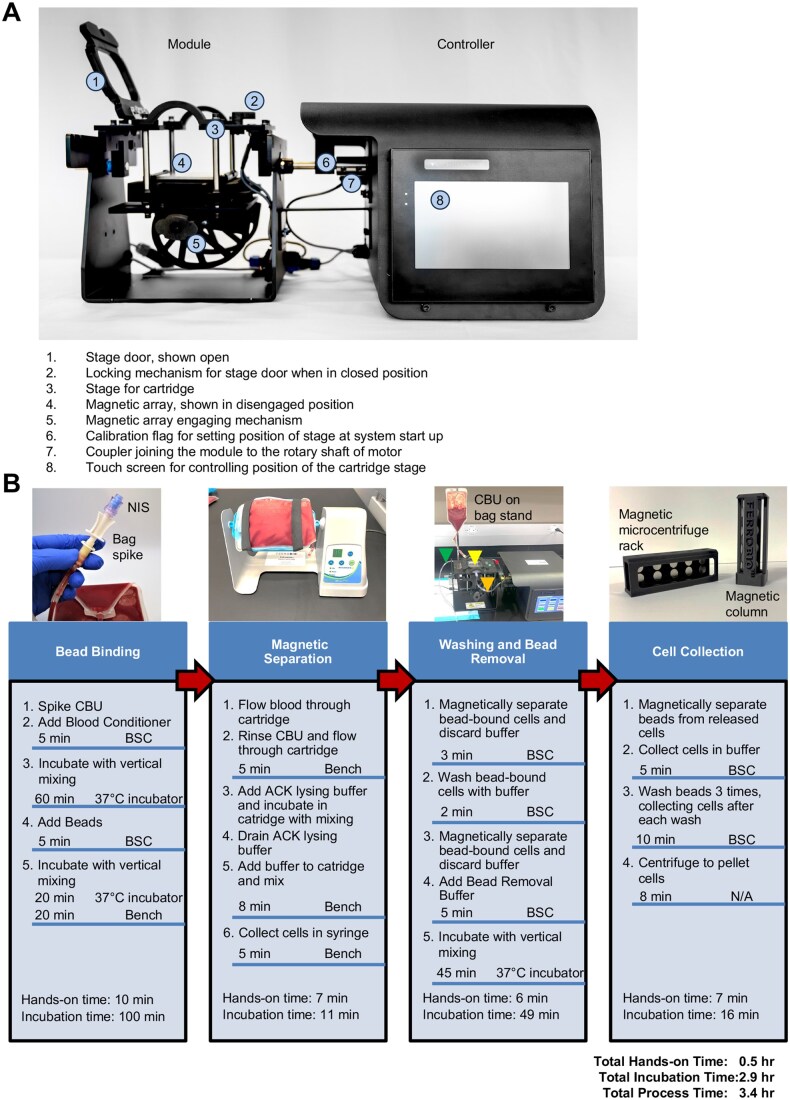
Overview of FerroBio™ hardware and isolation process. (A) Hardware: The FerroBio™ controller houses a motor that moves the module stage through various stationary positions and rock steps during protocol execution. The action of the motor is automated and controlled by the user with a touch screen. The cartridge is inserted into the module stage, and sits atop the magnetic array, which can be manually raised or lowered so that the magnetic field is in or out of range of the interior of the cartridge. (B) Process (from left to right): Bag spikes with self-closing, swabbable needless injection sites (NIS) are used with luer lock syringes to add blood conditioner and beads directly into the CBU and incubation steps take place on a Roto-mini mixer, or equivalent, with vertical mixing (end-over-end continuous rotation). After conditioning the blood and the completion of bead-binding, the CBU is mounted to the bag stand and blood flows by gravity through the inlet tubing (orange arrow) into the sterile FerroBio™ cartridge (yellow arrow), which is inserted into the module stage. With the magnet engaged, labeled CD34+ cells are separated, and all unlabeled blood components are drained from the cartridge through the outlet tubing (green arrow) into a sterile collection bag mounted on a bag stand below the device (not shown). Bead removal and final cell collection steps are performed manually with a micropipette in sterile, standard laboratory tubes using a custom magnetic column and microcentrifuge tube rack with a removable magnetic panel. Estimated times for each step are listed underneath the image and total time for the process is listed at the bottom. Hands-on time is defined as steps requiring direct interaction with reagents or equipment. Incubation time includes steps for conditioning, bead binding, bead removal, and magnetic separations during the bead removal and cell collection steps.

An overview of the FerroBio™ equipment is shown in Figure 1A and a summary of the key steps in the isolation procedure is shown in Figure 1B. Proprietary blood conditioner reagent (FB-4002) was reconstituted in Hank’s Balanced Salt Solution (HBSS) without phenol red, calcium, or magnesium to a concentration of 5 mg/mL and sterilized using a sterile PES syringe filter (Millipore-Sigma). Concentrated, reconstituted blood conditioner was added directly to the blood bag via a syringe connected to the bag spike to a final concentration of 0.1 mg/mL. CBUs were then fixed to the platform of a Roto-mini mixer (Fisher) using elastic bands and incubated at 37 °C for 1 h at 15 rpm vertical mixing (end-over-end). After incubation, FerroBio™ beads (FB-4001) were vortexed for thirty seconds to resuspend and added directly to the blood bag via a syringe connected to the FerroBio™ bag spike. The bead dose was based on initial CD34+ starting cell counts and estimated at about 50 beads per CD34+ cell. CD34+ cells were then labeled with beads by incubating the CBU for 20 min at 37 °C followed by 20 min at room temperature using the Roto-mixer as previously described.

#### Magnetic cell separation

After bead binding, CD34+ cells were separated from the rest of the blood components and washed using a semi-automated controller attached to a module (FB-1001), which houses the FerroBio™ cartridge (See Figure 1A for overview of FerroBio™ hardware). The FerroBio™ cartridge is situated atop a magnetic array that is built into the module and can be brought in and out of contact with the bottom of the cartridge. When engaged, the magnetic array generates a magnetic field within the cartridge to capture bead-bound cells as blood flows through the interior of the cartridge from the blood bag through a closed tubing system. Nontarget blood components, including un-labeled cells flow out of the cartridge and into an empty collection bag while the bead-bound cells remain in the cartridge. The CBU bag was then rinsed with HBSS containing 0.1% bovine serum albumin (BSA) to remove any remaining blood components and then flowed through the cartridge and into the collection bag. Bead-bound cells were then incubated for 5 min in ACK lysing buffer (Fisher) to remove contaminating red blood cells. The ACK lysing buffer was drained into the collection bag, and cells were then washed with HBSS + 0.1% BSA, removed from the cartridge via syringe, and transferred into a 50-mL conical tube.

#### Washing and bead removal

The 50-mL conical tube was placed into a custom FerroBio™ magnetic column for 2 min and the supernatant was removed. The bead-cell mixture was then washed in 2 mL of HBSS + 0.1%BSA and transferred to a 2-mL microcentrifuge tube and magnetically separated for 2 min using a custom FerroBio™ magnetic microcentrifuge tube rack, after which the supernatant was removed. FerroBio™ bead removal buffer was then added to the bead-cell mixture and diluted 1:2 with RPMI (with L-glutamine, but without HEPES, sodium pyruvate, or phenol red; Fisher), supplemented with 2% fetal bovine serum (FBS). Beads were then removed by incubating the mixture for 45 min at 37 °C in the Roto-mixer tube platform with vertical mixing.

#### Cell collection

After bead removal, the cell-bead mixture was magnetically separated for 2 min in the FerroBio™ magnetic tube rack, and the supernatant was collected in a 50-mL tube containing 9 mL RPMI + 2%FBS. Beads were washed and magnetically separated 3 additional times to ensure that all the cells were collected. The cell suspension was then magnetically separated for 2 min in the FerroBio™ magnetic column to remove any residual beads, and the cell suspension was transferred into a 15-mL conical tube.

### Cryopreservation

Following isolation cells were pelleted by centrifugation, analyzed for pre-cryopreservation quality metrics (see Supplementary Methods), and were cryopreserved.

### Immunophenotyping by cell surface staining and fluorescence-activated cell sorting

Freshly thawed (1 × 10^5^-1 × 10^6^) CD34+ enriched cells or cells from CD34+ expansion were incubated in PBS with fluorophore conjugated antibodies targeting CD34, CD38, CD45RA, CD10, CD49f, CD90, and CD135. Cells were washed 2x with cold PBS and were fixed with 1% paraformaldehyde (Pierce) and analyzed using FACS. Positive and negative gates were drawn based on unstained and single stained controls. Cell subpopulations were defined as follows: Hematopoietic Stem Cell (HSC): CD34+CD38-CD45RA-CD49f+CD90+; Multipotent Progenitor (MPP): CD34+CD38-CD45RA-CD49f-CD90-; Multilymphoid Progenitor (MLP): CD34+CD38-CD45RA+CD10+; Common Myeloid Progenitor (CMP): CD34+CD38+CD10-CD45RA-CD135+; Megakaryocyte Erythroid Progenitor (MEP): CD34+CD38+CD10-CD45RA-CD135-; Granulocyte Macrophage Progenitor (GMP): CD34+CD38+CD10-CD45RA+CD135+.

### *Ex vivo* colony forming unit assays

Colony forming unit (CFU)-GM, BFU-E, and CFU-GEMM colony numbers were derived by plating 250 freshly thawed CD34+ cells or 250 cells from CD34+ expansion assays in 1% methylcellulose/Iscove’s Modified Dulbecco’s Medium (IMDM) with 30% FBS (Corning), 1 U/mL recombinant human (rh) Epogen (EPO) (Amgen), 10 ng/mL rh Interleukin-3 (IL-3) (R&D Systems), 50 ng rh Stem cell factor (SCF) (R&D Systems), 10 ng rh Granulocyte/macrophage colony stimulating factor (GM-CSF) (R&D Systems), 2 mM L-glutamine, and 0.02 mM 2-Mercaptoethanol. Cultures were incubated at 37 °C in a humidified environment containing 5% CO_2_. Colonies were scored by manual counting 12 days after plating using an inverted microscope with phase contrast (Nikon) with a 4x objective.

### *Ex vivo* expansion assays

Thawed CD34+ cells were grown at a density of 5 × 10^4^ cells/mL in 1 mL SFEM II (STEMCELL Technologies) supplemented with 100 ng/mL recombinant human thrombopoietin (TPO), 100 ng/mL recombinant human Stem cell factor (SCF), and 100 ng/mL recombinant human FLT3 ligand (FLT3L) (R&D Technologies). Cultures were incubated at 37 °C in a humidified incubator kept at 5% CO_2_. On Day 4 after plating, fresh media containing the same growth factors was added to a total volume of 2 mL. On Day 7 after plating, cells were collected, nucleated cellularity was counted by Trypan Blue staining, and expanded cells were used for immunophenotyping or CFU assays.

### *In vivo* model of HSC/HPC potency

Twenty-four hours prior to transplantation, NSG mice were given a sublethal (350gy) whole body dose of gamma radiation. Irradiated mice were transplanted by tail vein injection with 500, 2500, or 10 000 freshly thawed CD34+ enriched cells (200 µL per injection). Doses were selected based on previous studies.^11^^,^^12^ Each group (CBU + Isolation Technique + Dose) contained 3 mice, for a total of *n* = 9 mice per cell dose and *n* = 3 CBUs per isolation technique. Peripheral blood (≤50 µL) was collected from each mouse every 4 weeks. Sixteen weeks following transplantation, mice were euthanized, and bone marrow was harvested by flushing. Flushed bone marrow (1 × 10^6^) or red blood cell depleted peripheral blood cells were stained with fluorophore conjugated antibodies: anti-CD45, anti-CD33, anti-CD19, and anti-CD3 for human chimerism and myeloid/lymphoid ratio analysis. Cells were washed 3 times, fixed, and analyzed by FACS. Mice with greater than twice the amount of human chimerism compared to an untransplanted control were considered engrafted, and Extreme Limiting Dilution Analysis (ELDA)^13^ was performed to determine SCID repopulating cell (SRC) frequency.

### Electron microscopy

Detailed methods are described in the Supplementary Materials. Briefly, one million freshly thawed CD34^+^-enriched cells were processed per sample. Cells were first fixed with glutaraldehyde and paraformaldehyde, washed, and subjected to a second fixation step. Samples were, then, dehydrated through a graded ethanola, infiltrated with resin, embedded, and ultrathin‑sectioned for TEM imaging. Bead-only controls were processed in parallel to visualize microbeads. TEM was performed using a Tecnai Spirit BioTwin (FEI, Hillsboro, OR, USA) at 80 kV with an AMT NanoSprint 6 CMOS camera (AMT, Woburn, MA, USA).

## Results

### FerroBio™ is a streamlined human CD34+ isolation technology

Recently, FerroBio™ was developed as a novel cell isolation technology by FerroBio™ Technologies. In this study, we present a comparison of column-based isolation of CD34+ cells with FerroBio™ technology. Figure 1A shows an overview of the FerroBio™ hardware. The technology allows for streamlined, semi-automated isolation of CD34+ cells by using a touch screen to move the cells through various stages of isolation. The hardware consists of a stage where the FerroBio™ cartridge is loaded, a magnetic array that allows for isolation of immunomagnetically tagged CD34+ cells, and a motor that moves the sample through different stages throughout protocol execution. Figure 1B shows an overview of the standard protocol for isolating CD34+ cells using FerroBio™ (details in Methods). Briefly, CBUs are conditioned for 60 min with gentle mixing with proprietary buffer, then are incubated with immunomagnetic beads with gentle mixing for 40 min (20 min each at 37 °C and ambient temperature). Blood is flowed through the FerroBio™ cartridge and bead-tagged cells are separated from the rest of the blood components by the magnetic array. Isolated cells are washed, rinsed with ACK red blood cell lysis buffer and washed again. Beads are released from the cells and bead-free cells are collected manually with a micropipette for downstream use. In all, the hands-on time for this procedure is approximately 0.5 h and the total processing time is less than 3.5 h.

### FerroBio™ achieves high overall CD34+ purity

**Figure 2.**
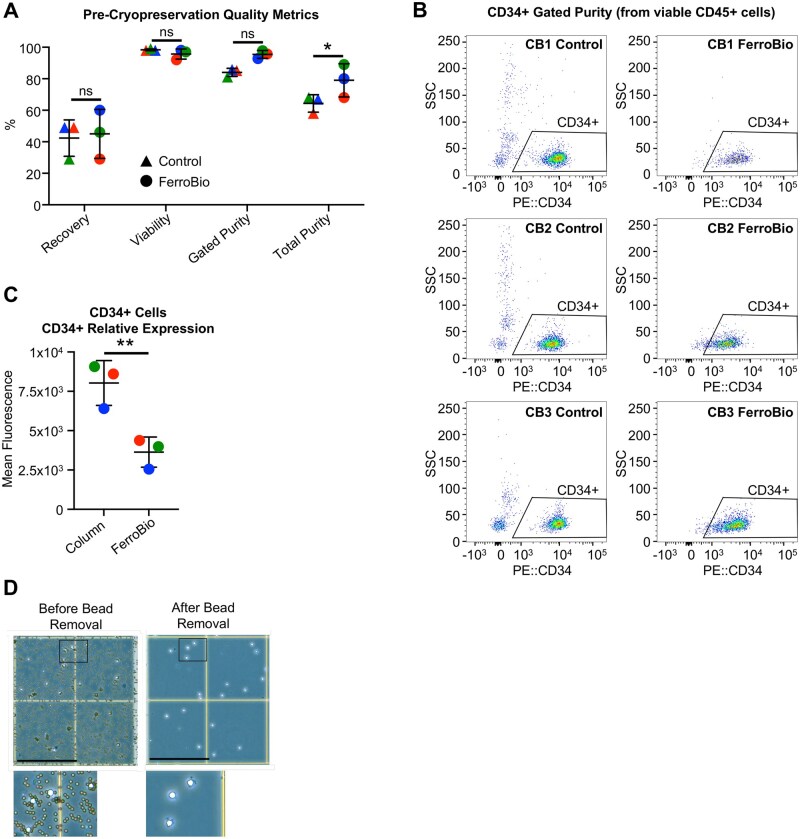
Pre-cryopreservation viability and purity of FerroBio™ isolated CD34+ cells. (A) Percent recovery, viability by 7-AAD staining, gated purity and total purity of freshly isolated cells (calculated as CD34+cells/total nucleated cells) wer measured by flow cytometry. (B) Representative flow cytometry plots of fresh cell isolates from each CBU before cryopreservation. (C) Mean fluorescence intensity for CD34 expression within the CD34+ population of cells. (D) Microphotographs of cells isolated with FerroBio™ or control before cryopreservation at 100x magnification (phase contrast, scale bar = 250 µm). Inset: Zoomed in image of area delineated by black box. Stats: paired *t*-tests matching for CBU. ns = not significant, *P < .05. Each different colored point represents a different CBU.

To test whether different CD34+ cell isolation techniques affect cell potency, we performed a side-by-side comparison between FerroBio™ and a column-based isolation technique. Whole blood volume from non-cryopreserved freshly harvested CBUs isolated within 55 h (Table 1) was split into two fractions. One half of the blood was subjected to CD34+ isolation using column-based technique while the other half was subjected to CD34+ isolation using FerroBio™. The quality of the cell isolates was assessed directly after isolation. CD34+ cell recovery, viability, and gated purity were not statistically different between isolation methods (Figure 2A). Total purity was significantly higher in FerroBio™ isolates compared to control prior to cryopreservation based on the percentage of total nucleated cells that were CD34+ (Figure 2A). The presence of non-target cells in control isolates was also evident in flow cytometry scatter plots, where CD45+CD34- cells with variable side scatter properties were visible in control isolates but largely absent in cells prepared with FerroBio™ (Figure 2B). Interestingly, CD34+ cells isolated by FerroBio™ have significantly lower mean fluorescence intensity for cell surface CD34 expression (Figure 2C), which is maintained following cryopreservation and thaw (data not shown). This may be caused by technical artifacts from using different antibodies for isolation or from the residual presence of beads in the control group. This could also reflect biological changes, though the effects of variable levels of CD34 expression within the CD34+ population are not well-understood. Thus, we will next test differences in functional potency in cells from the two different isolation techniques. The removal of beads on the CD34+ cells isolated by FerroBio™ were confirmed using phase contrast light microscopy at 100× magnification (Figure 2D). Following this “split-bag” isolation, CD34+ cells were cryopreserved.

**Table:** 

Table 1. Cord blood unit preprocessing attributes.						
CBU number	Baby sex	Ethnicity	Total nucleated cells	Total CD34 cells	Volume (mL)	CBU age at testing (h)
**1**	Male	White	9.50 E + 08	3.35 E + 06	101.1	48.7
**2**	Female	White	1.86 E + 09	3.00 E + 06	139.7	50.8
**3**	Male	Black	7.17 E + 08	4.65 E + 06	93.6	22.9

### Isolation with FerroBio™ yields increased post-cryopreservation HSC numbers

**Figure 3.**
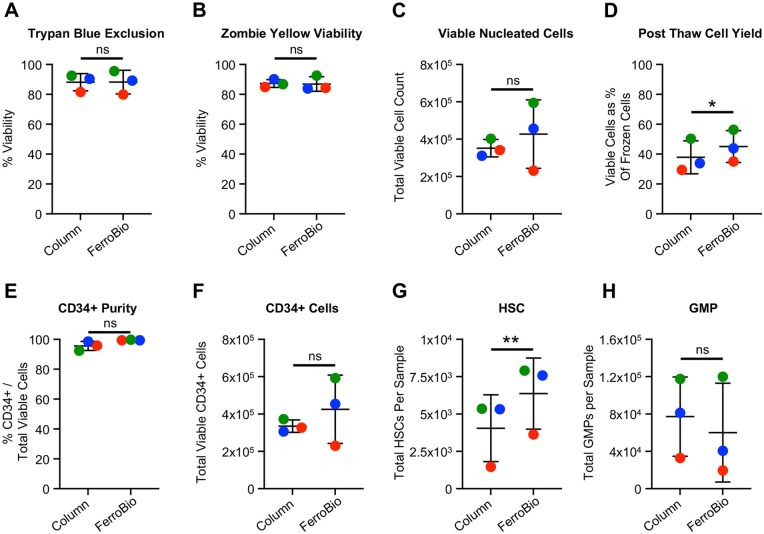
Post-cryopreservation Immunophenotyping of FerroBio™ isolated CD34+ cells. (A-H) Following cryopreservation and thawing, CD34+ cells from FerroBio™ isolations or column-based control isolations were counted and stained with antibodies targeting cell surface markers for immunophenotyping by flow cytometry. Viability percentage by (A) Trypan Blue Staining or (B) Zombie Yellow fixable viability dye negativity. (C) Total nucleated viable cells following thaw. (D) Percent yield of total nucleated cells compared to the number cryopreserved. (E) CD34+ purity post-thaw. (F-H) Total number of the indicated immunophenotypic HSC/HPC subpopulation following thaw. Each different colored point represents a different CBU. Stats: paired *t*-tests matching for CBU. ns = not significant, *P < .05, **P < .01.

Cryopreserved CD34+ cells from the “split-bag” isolation procedures were thawed rapidly and washed of cryopreservation media. Following thaw, cells were first analyzed for overall recovery. By two separate viability staining methods (Trypan Blue with hemacytometer counting and fixable viability dye staining followed by flow cytometry), there were no significant differences in the percent of live cells between the traditional column isolation and the FerroBio™ isolation (Figure 3A and B). There were also no significant differences in total viable nucleated cell count (Figure 3C), but FerroBio™ isolation yielded slightly but significantly better percent recovery of viable cells when compared to the number of cells frozen in each sample (Figure 3D).

Next, CD34+ enriched cells from FerroBio™ or column-based isolations were stained with fluorescently conjugated antibodies targeting cell surface proteins for the identification of specific HSC/HPC subpopulations by flow cytometry (Supplementary Figure S1).^2^ This post-thaw immunophenotyping showed that FerroBio™ had statistically similar numbers and purity of CD34+ cells (Figure 3E and F) and showed no differences in frequencies of immunophenotypically defined HSCs/HPCs (Supplementary Figure S2A-C). Interestingly, we found that FerroBio™ yielded significantly higher total numbers of post-thaw HSCs compared to control (Figure 3G). Given the similar frequency of HSCs but the increased total cell yield and HSC yield, this difference is likely due to HSCs being particularly sensitive to freeze-thaw processes, which may be partially mitigated by isolation with FerroBio™. We also observed statistically similar numbers of CD34+CD38- primitive progenitors, CD34+CD38+ mature progenitors, MPPs, MLPs, CMPs, MEPs, and GMPs in both isolation methods (Figure 3H and Supplementary Figure S2E–I).

### Isolation with FerroBio™ yields cells with higher potent cell expansion capacity

**Figure 4.**
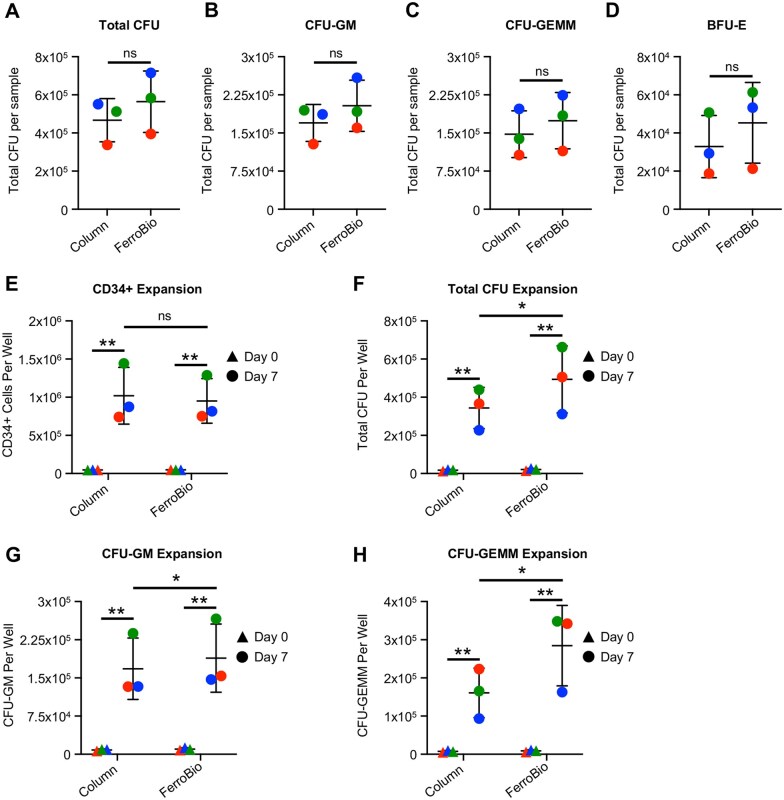
CFU and expansion capacity of FerroBio™ isolated cells. (A–H) Following cryopreservation and thawing, CD34+ cells from FerroBio™ isolations or column-based control isolations were (A–D) plated in semisolid methylcellulose with growth factors for 12 days and counted for enumeration of the indicated CFUs or (E–H) plated in liquid expansion cultures with growth factors and then subjected to immunophenotyping for the indicated population or CFU assays for the indicated CFU type. Stats: paired *t*-tests matching for CBU. ns = not significant, *P < .05, **P < .01. Each different colored point represents a different CBU.

To test for differences in *ex vivo* HPC potency, we analyzed the post-thaw CFU capacity of CD34+ cells isolated using the two different techniques. There were no significant differences in the total number of CFU, CFU-GEMM, CFU-GM, or BFU-E between FerroBio™ and control, though all trended toward increased CFU capacity in cells isolated using FerroBio™ (Figure 4A–D). Next, we examined the proliferative, self-renewal, and differentiation capacities of CD34+ cells *ex vivo* using expansion assays. CD34+ cells were cultured in serum free media with TPO, FLT3L, and SCF growth factors for 7 days. CD34+ expanded significantly in culture in both groups. Immunophenotyping of expanded cell populations did not show significant differences in any examined HSC/HPC subpopulations between the isolation groups (Figure 4E and Supplementary Figure S3A–F). We also examined CFU expansion by plating expanded cells in methylcellulose media and monitoring colony growth. Interestingly, there was significantly increased total CFU expansion in the FerroBio™ group, and FerroBio™ isolated cells yielded higher numbers of CFU-GM and CFU-GEMM subtypes after 7 days of expansion (Figure 4F–H). BFU-E did not expand in either condition. This is consistent with FerroBio™ trending toward higher CFU capacity immediately post-thaw, and may indicate improved HPC function. In all, these data suggest that FerroBio™ can be used to isolate HSCs/HPCs for *ex vivo* analyses consistent with commonly used technology.

### FerroBio™ yields human CD34+ cells with *in vivo* potency

**Figure 5.**
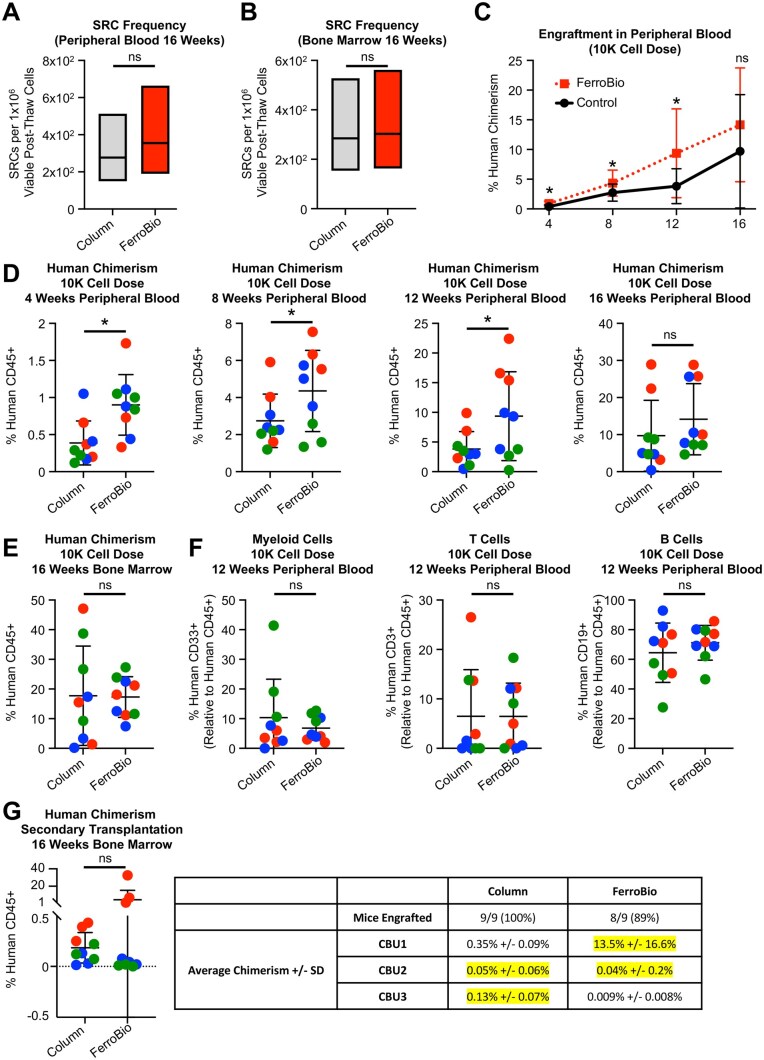
*In vivo* repopulating capacity of FerroBio isolated cells. (A-F) Sublethally irradiated NSG mice were transplanted with three doses of CD34+ cells from FerroBio isolations or column-based control isolations. (A, B) SCID repopulating frequencies (SRCs) in peripheral blood and bone marrow of mice 16 weeks post-transplantation. (C) Peripheral blood engraftment over time of the highest cell dose. (D) Peripheral blood engraftment at the individual time points of the highest cell dose. (E) Bone marrow engraftment at the 16-week endpoint of the highest cell dose. (F) Myeloid and lymphoid cell composition as a percentage of total human CD45+ cells in peripheral blood of mice at week 12 post-transplantation. (G) Secondary engraftment assays were performed by pooling bone marrow from primary recipients receiving the highest dose of cells and transplanting 5 × 10^6^ cells to secondary sublethally irradiated recipient mice. Shown is the bone marrow chimerism at week 16 following secondary transplantation. Also shown are the frequency of engrafted mice and average chimerism. Stats: SRC frequency was compared by limdil linear modeling in R. Engraftment was compared at each individual time point comparing isolation techniques and controlling for CBU as a random effect using linear modeling and ANOVA. ns = not significant, *P < .05. Each different colored point in D-F represents a different CBU.

For a cell isolation technique to be viable for use in the field of CD34+ cell research, it is critical that HSCs/HPCs retain *in vivo* potency following processing.^3^^,^^12^^,^^14^ A gold standard metric for human HSC/HPC potency is the ability to repopulate a human hematopoietic system in immune deficient mice. To that end, we transplanted post-thaw CD34+ cells isolated by FerroBio™ or column-based kits at three serially diluted cell doses (500, 2500, and 10 000 cells) to analyze differences in engraftment and in engraftable cell frequency (Supplementary Figure S4A–E). Mice were monitored for human chimerism in the peripheral blood every 4 weeks until week 16, at which point they were euthanized and bone marrow chimerism was also analyzed. We first found that FerroBio had statistically similar SCID repopulating cell (SRC) frequency using limiting dilution linear modeling at all time points in either peripheral blood or bone marrow (Figure 5A and B and Supplementary Figure S5A–C). Interestingly, we found significantly higher peripheral blood chimerism in mice transplanted with the highest dose of FerroBio™ cells at early and intermediate engraftment times (weeks 4, 8, and 12) compared to control (Figure 5C and D). Peripheral blood and bone marrow showed no difference in long-term engraftment at week 16 (Figure 5C–E) or at any time point for lower cell doses (Supplementary Figure S5D and E). There were no significant alterations in myeloid to lymphoid ratio as measured in the peripheral blood at weeks 8 and 12 (Figure 5F and  Supplementary Figure S5F). To test long-term fitness of the HSCs, we performed secondary engraftment assays where bone marrow from the primary recipient mice receiving the highest dose of cells were transplanted to secondary recipient mice. All except one mouse (from the FerroBio™ group) exhibited secondary engraftment, with one CBU showing better engraftment for each isolation condition (Figure 5G). Thus, there are no significant observable differences in long-term HSC fitness. These data suggest that FerroBio™ isolation did not give significantly altered frequencies of engraftable cells or lineage skewing and may yield more rapid generation of humanized hematopoietic systems in mice when delivered at a sufficient cell dose.

### FerroBio™ removes beads that may impact cell health

**Figure 6.**
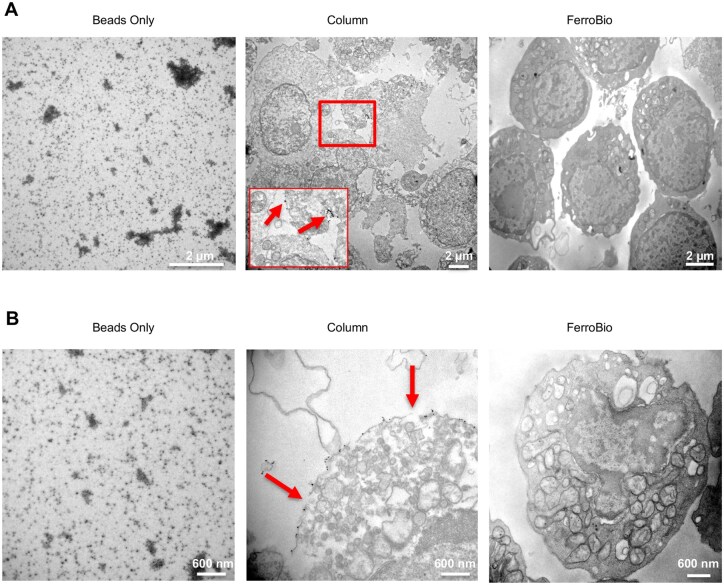
Transmission electron microscopy of FerroBio isolated cells. (A) 4800x or (B) 23 000x magnification of cells from the indicated isolations. Arrows are pointing to microbeads.

We hypothesized that the differences we observed in function may be due to the persistence of magnetic micro beads following isolation with column-based kits. We, thus, performed transmission electron microscopy (TEM) to evaluate the presence of these beads, observe their localization, and note changes to cellular morphology (Figure 6A and B). To achieve the proper cell density, this analysis was performed post-cryopreservation-thaw on separate CBUs where the full volume was subjected to either FerroBio™ isolation or column-based kit isolation. Following thaw, we found that most cells in the FerroBio™ group displayed normal morphology (clearly visible intracellular organelles with outer cell membranes intact and continuous). In contrast, there was a higher frequency of cells from the column-based kit isolation displaying distorted morphology and discontinuous or degraded cell membranes. Magnetic microbeads were clearly visible in the column-based kit isolated cells and not in the FerroBio™ group. Notably, the cells that displayed unhealthy appearance were coated in the magnetic microbeads at a high density (Figure 6A and B). This suggests that cells that are highly tagged with the immunomagnetic beads may be more susceptible to stress induced cell death following cryopreservation and thaw. If the microbeads indeed affect cell health, this could be one cause of some of the modest differences observed in yield, purity, recovery, and potency between the FerroBio™ isolation group and cells from the column-based kits.

## Discussion

In this study, we compared the *ex vivo* and *in vivo* properties of CD34+ cells isolated using the novel FerroBio™ technology compared to a control column-based approach. The column-based approach utilized in this comparative study is designed for isolation of ultra-pure CD34+ cells and is considered a high standard isolation technique (compared to other techniques that may produce suboptimal purities). FerroBio™ isolation yielded similar numbers of viable CD34+ cells with slightly higher total purity compared to control. FerroBio™ isolation also yielded similar distributions of HPCs compared to control but did contain a significantly higher number of HSCs. *Ex vivo* functional analyses suggest that FerroBio™ isolated CD34+ cells have similar colony-forming capacity, but higher *ex vivo* expansion capacity compared to cells isolated with control kits. Interestingly, cells isolated with the FerroBio™ technique resulted in higher human chimerism in the peripheral blood of NSG mice 4 through 12 weeks after injection compared to the control method and trended towards faster engraftment rates at the highest cell dose. In all, the findings from this study suggest that the isolation technique used to enrich CD34+ cells play a role in the yield, purity, recovery, subpopulation composition, *ex vivo* expansion, and *in vivo* potency of the cells.

Two critical parts of traditional column-based isolation procedures include a long, relatively high-speed density gradient centrifugation and immunomagnetic labelling of CD34+ cells.^10^^,^^11^ The spin required for density centrifugation is often higher speed and more than three times as long as is typically required to pellet purified CD34+ cells and may, thus, induce mechanical stress. Indeed, we have observed that cells that are particularly sensitive, ie, whole cryopreserved blood immediately following thaw, are more likely to lyse during density centrifugation compared to other methods such as red blood cell depletion (unpublished observations). Layering cells on density gradients and collection of the low-density fraction is also time consuming and technically challenging, which may lead to slow and inefficient isolation. Additionally, increased wash steps for column-based strategies may increase cell stress and processing time. Streamlined workflows like FerroBio™ with parallel module technology that allows for processing many CBUs concurrently potentially reduces the difficulty and time of processing compared to density gradient and column-based isolations, although, here, we did not directly compare processing times.

Similarly, the persistence of microbeads on the surface of cells following immunomagnetic separation may affect cell health and function. In this study, we observed changes in cell morphology reflective of health correlated with the presence of magnetic microbeads. Previous studies have also shown that *in vitro* proliferation is affected by persistence of magnetic microbeads on the cell surface, though the frequency of long-term initiating cells and CFUs were unaffected.^9^^,^^15^ In those previous studies, freshly isolated CD34+ cells from bone marrow showed clusters of beads in roughly half of the isolated cells, which were localized to the membrane, typically at the poles of the cells or in intracellular vesicles. Beads were however absent in 70% of the CD34-enriched cell isolate after 48 h of incubation in cell culture medium suggesting that the cells either degrade or expel the beads. Interestingly, T-cells isolated from peripheral blood using immunomagnetic isolation had persistent intracellular and membrane-bound beads and maintained magnetic properties for up to 2 weeks when cultured in non-proliferating conditions,^15^ suggesting dormant or quiescent cells may be more likely to retain these beads. As quiescence is an important characteristic of functional HSCs, this is noteworthy. One study even demonstrated that intramyocardially injected stem cells were positive for presence of beads after transplantation, though the beads appeared to be lost by 48 h post-transplantation.^16^ Finally, passing cells through a column matrix and eluting by mechanical force may cause additional mechanical or shearing stress to the cells. Thus, the persistence of magnetic microbeads, density gradient centrifugation, and mechanical stressors may be confounding variables affecting cell health both *ex vivo* and *in vivo* and should be considered when isolating cells for research or clinical use. These confounding effects may explain at least in part the differences we found in cell recovery and potency in FerroBio™ isolated cells compared to column-based kits.

Here, we present data that should be considered when selecting isolation techniques for the use of *ex vivo* or *in vivo* modeling of HSC/HPC function. For example, if immunophenotypic HSCs or cells with higher colony forming capacity are the target population for a given study, our data suggests that bead-free CD34+ cell isolations may be better for maintenance of these cell types. As another example, immunodeficient mice with humanized immune systems are a gold standard preclinical model in immune-oncology, stem cell biology, and infectious disease research. Considerable effort has been made to improve mouse models of the human immune system using different immunodeficient mutant mouse strains,^17–21^ co-injection with human cytokines,^22^ or CD34- cells,^23^ different CD34+ injection sites,^24^ and utilizing mice of various ages (newborn or adult mice).^25^ Interestingly, variable engraftment characteristics have been described among cells isolated from cord blood, bone marrow, or mobilized peripheral blood. In this study, our data suggests that bead-free CD34+ cells may yield more rapid and/or predictable engraftment in immune deficient mice. In some instances, these differences are subtle, but they could be biologically significant when dealing with donor sources that are already limited by cell number and volume. Thus, isolation technique should be taken into account when planning the generation of humanized immune systems in mouse models, and specific research applications should be tested following different isolation methods with increased sample sizes.

CD34+ cells, particularly those derived from umbilical cord blood, are of increasing importance for clinical translation. CD34+ expansion in particular has shown promise as an option to expand access to transplantation to those underrepresented on adult hematopoietic cell registries. Additionally, the derivation of specific cell populations like T-cells or NK- cells for use in immunotherapies from umbilical cord blood HSCs/HPCs may allow for development of future off-the-shelf therapies.^3^^,^^26–31^ Indeed, CAR-T cells derived from HSCs have shown promise in preclinical studies.^29^ For these uses, the potency of the HSCs/HPCs is critical, and isolation technique may be one factor affecting whether a unit is usable for these purposes or not. Finally, maintaining cell health may also be important for using CD34+ cells from bone marrow or peripheral blood donor sources for research purposes or clinical uses like gene editing.^32^^,^^33^ Future studies are warranted to determine whether density gradient free isolations resulting in bead free isolates like FerroBio™ are viable options for the isolation of clinical grade cell therapies and whether other donor sources achieve similar metrics as umbilical cord blood using these systems. It will be critical for each separate clinical application to test differences achieved in efficiency and efficacy through the utilization of different isolation techniques. In all, the data presented in this study provides researchers and those seeking improved methods by which to prepare CD34+ based clinical products a direct comparison of commonly used isolation techniques with the novel FerroBio™ approach.

## Limitations of study

One limitation of this study is that given the proprietary nature of the CD34+ antibody used in FerroBio, we cannot directly assess if differences in purity are due to antibody affinities or other factors. An additional limitation is the low sample size. The donor-matched comparison should decrease the confounding factors typically associated with studying primary human samples (which usually require higher *n* for accurate comparison of unmatched samples), but the sample size remains a limiting factor. It will be critical that isolation techniques are directly compared for any proposed application (ie, CB CD34+ expansion for transplantation in the clinic) with increased sample sizes.

## Supplementary Material

szaf067_Supplementary_Data

## Data Availability

The data underlying this article are available in the article and in its online Supplementary material.
