# Supplementary material for: Potency of human hematopoietic cells from a novel CD34+ isolation technique
Source: Stem Cells Transl Med. 2025 Dec 10;14(12):szaf067. doi: 10.1093/stcltm/szaf067 (PMC12688439; doi:10.1093/stcltm/szaf067)
Supplement: szaf067_Supplementary_Data [file szaf067_supplementary_data.pdf]

Supplemental Information

Potency of Human Hematopoietic Cells From a Novel CD34+ Isolation Technique

James Ropa[1]*, Jimin Park[1], Jessica Newton[1], So Jeong Kim[1], Yangshin Park[2], Jonathan Messer[3], Justin Blacher[3], and Shabnam Namin[3]

[1]Department of Medical and Molecular Genetics, Indiana University School of Medicine, Indianapolis, IN 46202

[2]Department of Biochemistry and Molecular Biology, Indiana University School of Medicine, Indianapolis, IN 46202

[3]42Bio Inc., Gainesville, FL 32601

*Correspondence to: James Ropa, Indiana University School of Medicine, 975 W Walnut St IB 454B, Indianapolis, IN, 46202; tel: 317-278-6364; email: jropa@iu.edu

Supplemental Materials and Methods

Cord Blood Acquisition and Pre-Isolation Analysis

Donated cord blood units (n=3 CBU) for research use were acquired from two independent blood banks, one in north central Florida and the other in Ohio. Incoming CBU attributes were established by the blood banks prior to CD34 isolation. Total CBU volume (umbilical cord blood plus 35 mL of anti-coagulant) was estimated based on collection bag weight and blood density. Total nucleated cells and CD34+ cells were enumerated with automated cell counting and BD StemCell Enumeration Kit (BD Biosciences) for flow cytometry, respectively. Viability was assessed by 7-AAD staining. Flow cytometry input was gated based on ISHAGE Guidelines [13]. Blood age at testing was calculated as the time elapsed between cord blood collection and the addition of FerroBio™ Beads. See Table I for CBU attributes for this study.

CD34+ Cell Isolations and Pre-Cryopreservation Analysis

All reagents for each isolation technique were purchased pre-sterile or were sterile filtered prior to use. Open tubes containing cells were handled within a biosafety cabinet to avoid microbial contamination. To eliminate donor variability, cord blood was evenly split by volume. To split blood, a bag spike was inserted into the CBU bag port, and blood was removed via syringe through a swabbable needleless injection site attached to the spike. Blood was then aliquoted into 50 mL conical tubes for the column-based control isolation, and the remaining volume was left in the CBU collection bag for the FerroBio™ isolation. After splitting, each isolation technique was performed concurrently to eliminate the potential influence of storage temperature and blood ageing. At the end of each isolation procedure, samples were taken for pre-crypreservation analysis and the remaining cells were suspended at ≤1E+06 cells/mL in fetal bovine serum supplemented with 10% dimethyl sulfoxide. Cryovials of cells were put into Mr. Frosty Cryopreservation Containers (Fisher Scientific) and frozen at -80°C before moving into long term storage at -135°C. Samples of freshly isolated cells from each isolation technique were submitted to a CLIA-certified cGMP blood bank laboratory for analysis. Total nucleated cell counts were calculated with an automated Sysmex XN-10 cell counter. CD34+ cells were counted using BD StemCell Enumeration Kit using a BD FACSLyric cytometer with BD FACSuite Clinical software (v1.5) with viability assessment and gating strategy as described above. Total purity was estimated by dividing the total number of CD34+ cells by the number of total nucleated cells in the isolates.

Statistics

Statistical analysis was performed using GraphPad Prism (version 10.4.2). Data are presented as mean ± standard deviation. P-values less than 0.05 were considered statistically significant. Paired t-tests with CBU matching were used to determine significant differences between isolation techniques for recovery, viability, gated purity, and total purity in freshly isolated cells. Two-way ANOVA with post-hoc Sidak’s test was used to confirm expansion and assess changes in expansion at Day 7 between the two isolation techniques. Mixed effects linear modeling with ANOVA was used to analyze human chimerism in transplanted mice at each time point. Models were corrected for

CBU batch effects by making CBU the random effect and isolation technique the fixed effect.

Electron microscopy
One million cells per sample were pelleted at 500 ×g for 7 min at 4°C. After decanting the supernatant, the pellets were fixed in 1 mL of 0.1M cacodylate buffer (pH 7.4) containing 2.5% glutaraldehyde and 2% paraformaldehyde (EM Sciences, Hatfield, PA, USA) and incubated overnight at 4˚C. The fixed pellets were washed six times with 1 mL of 0.1M cacodylate buffer (pH 7.4). Secondary fixation was performed by adding 1 mL of 0.1M cacodylate buffer (pH 7.4) containing 1% OsO_4 and 1.5% potassium ferrocyanide (EM Sciences, Hatfield, PA, USA) and incubated at room temperature for 1 hour. The fixed pellets were washed three times with 1 mL of 0.1M cacodylate buffer (pH 7.4). The samples were dehydrated at room temperature with 3 mL aliquots of a series of ethanol solutions: 30% ethanol for 10 min; 50% ethanol for 20 min; 70% ethanol for 20 min; two changes of 95% ethanol for 15 min each; and two changes of 100% ethanol for 30 min each. The samples were then infiltrated at room temperature on a rotator (Thermo Fisher Scientific, Waltham, MA, USA) set to 60 rpm with 3 mL aliquots of a series of SPURR resin (EM Sciences, Hatfield, PA, USA) solutions: one part SPURR resin and two parts 100% ethanol for 12-hours; one part SPURR resin and one part 100% ethanol for 24-hours; three parts SPURR resin and one part 100% ethanol for 3 hours; 100% SPURR resin for 8 hours. The cell pellets were then embedded in a Double End Tapered Embedding Mold (Ted Pella Inc., Redding, CA, USA) filled with 350 µL of fresh SPURR resin. The embedding resin was polymerized by incubating for 12 hours at 70˚C in a bench-top incubator (VWR, Randor, PA, USA). Ultrathin sections (70 nm) were cut using an ultramicrotome (Leica Microsystems, Wetzler, Germany) with a diamond knife (Diatome, Quakertown, PA, USA) and placed on 200 mesh nickel grids (EM Sciences, Hatfield, PA, USA). The sections were dried in a vacuum desiccator (SP BelArt, Wayne, NJ, USA) for 1 hour before imaging.

The magnetic beads were processed similarly as the cells, however, the unbound beads did not form a pellet after centrifugation. Therefore, the solution beads was used directly for imaging. 5 µL of beads was placed on 200 mesh nickel grids (EM Sciences, Hatfield, PA, USA) and incubated at room temperature for 1 min. The grid was then wicked dry with the tip of a piece of Whatman filter, then washed with one drop of deionized water. Stained with 2% uranyl acetate (EM Sciences, Hatfield, PA, USA) for 1min, then wicked and washed as above and let dry at room temperature.

Micrographs were collected on a Tecnai Spirit BioTwin transmission electron microscope (FEI, Hillsboro, OR, USA) operated at 80 kV with an AMT NanoSprint 6 CMOS camera (AMT, Woburn, MA, USA).

Supplemental Figures

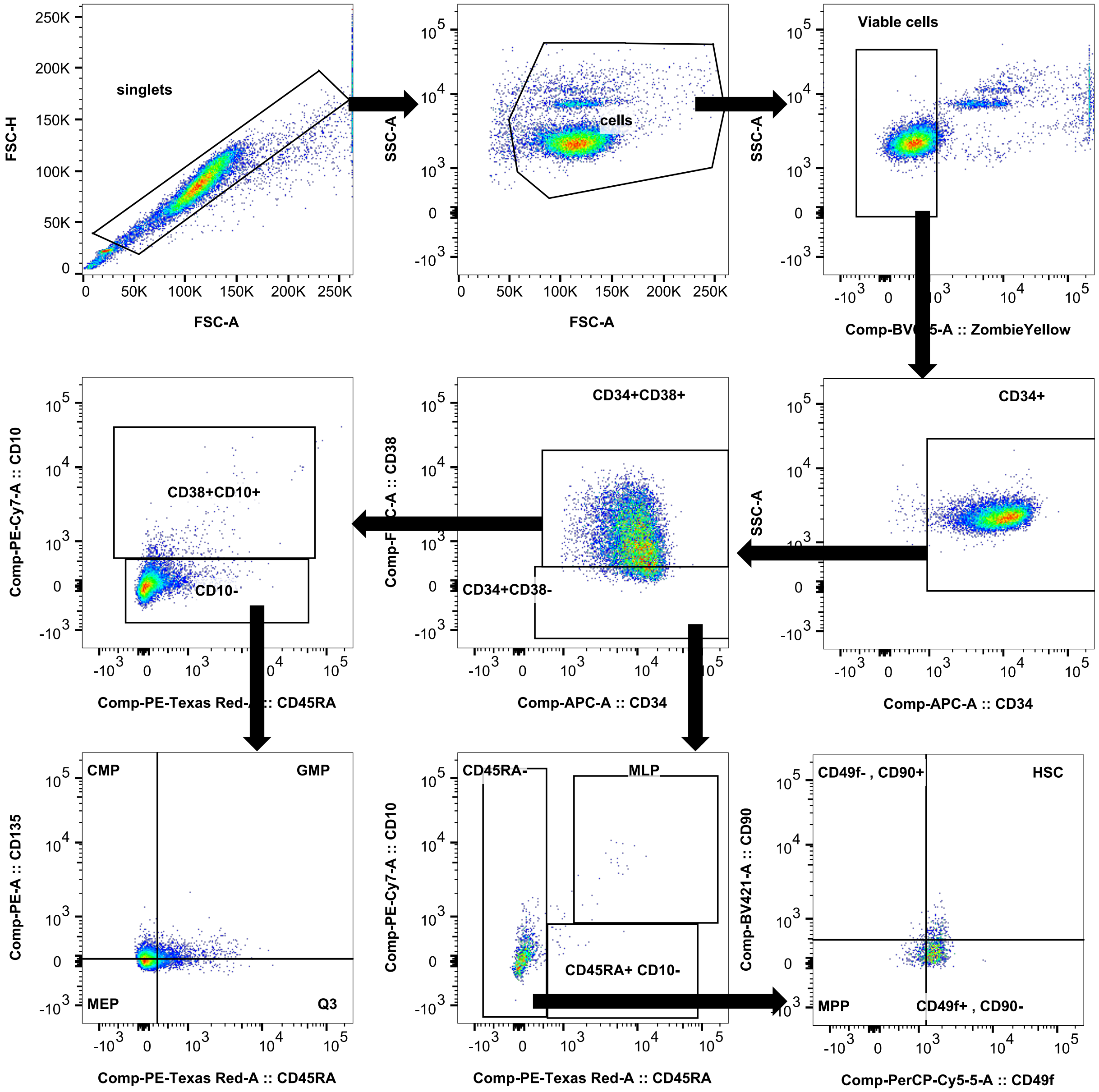

Supplemental Figure 1 (related to Figure 3). Gating Strategy for identifying immunophenotypically defined HSCs/HPCs subpopulations.

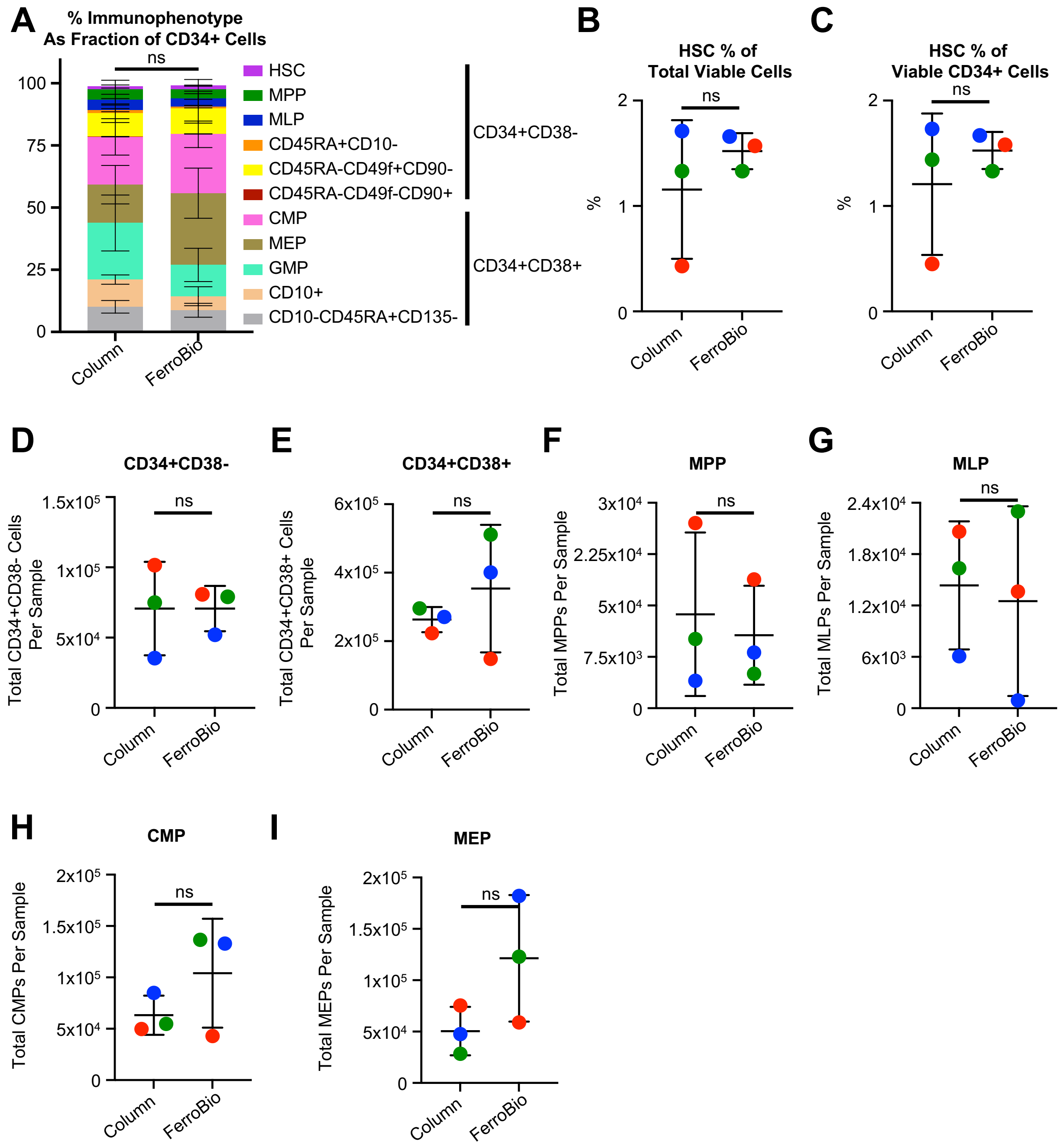

Supplemental Figure 2 (related to Figure 3). A) Percentage of CD34+ cells that were immunophenotypically defined as the indicated cell populations. B-C) Representative plots of B) % of total viable cells or C) % of CD34+ cells that were defined by the indicated immunophenotype, in this case HSCs. D-I) Following cryopreservation and thawing, CD34+ cells from FerroBio™ isolations or column-based control isolations were counted and stained with antibodies targeting cell surface markers for immunophenotyping by flow cytometry. Total number of the indicated immunophenotypic HSC/HPC subpopulation following thaw. Each different colored point represents a different CBU. Stats: paired t-tests matching for CBU. ns = not significant.

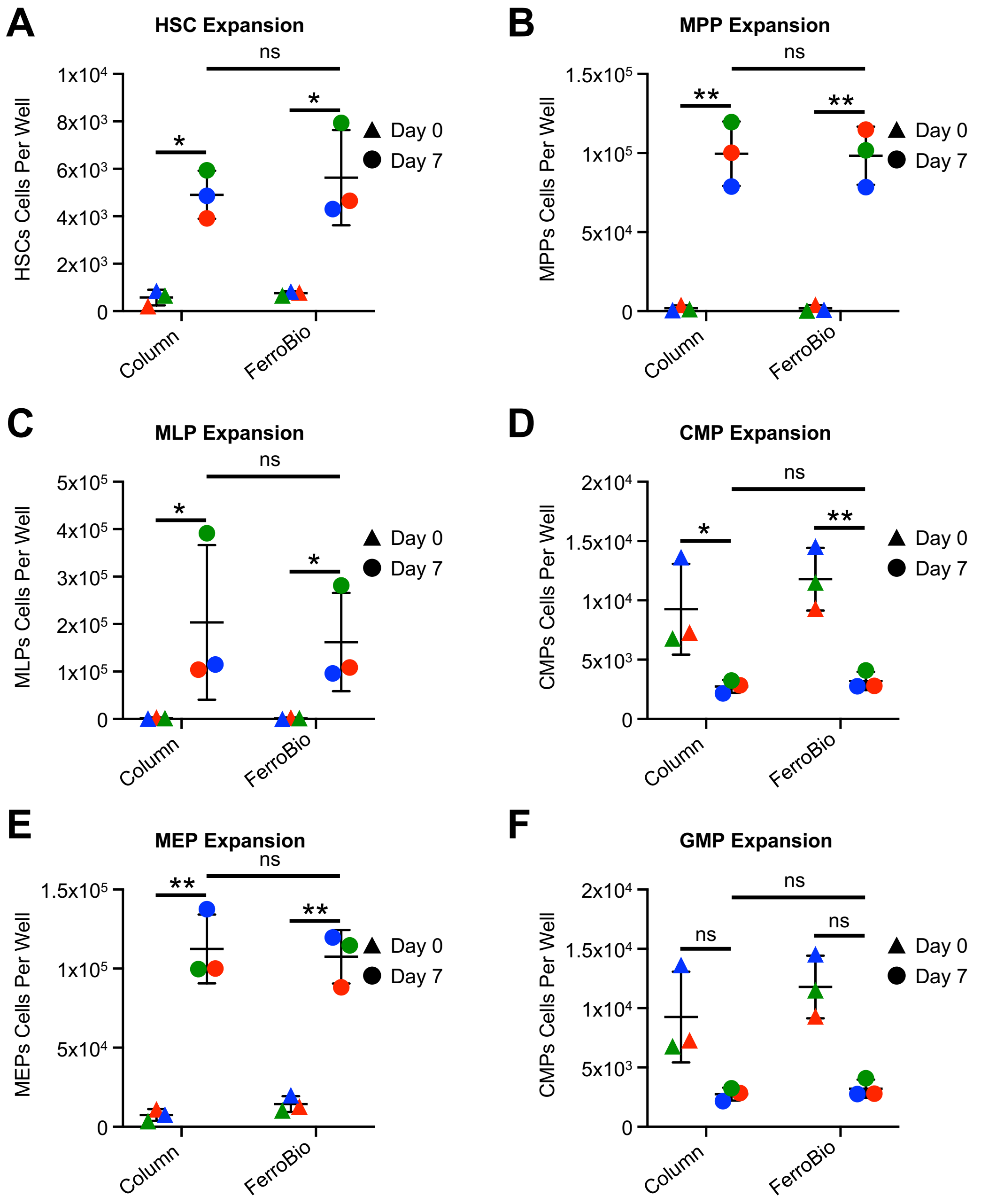

Supplemental Figure 3 (Related to Figure 4). A-F) Following cryopreservation and thawing, CD34+ cells from FerroBio™ isolations or column-based control isolations were plated in liquid expansion cultures with growth factors and then subjected to immunophenotyping for the indicated population. Stats: paired t-tests matching for CBU. ns = not significant. Each different colored point represents a different CBU.

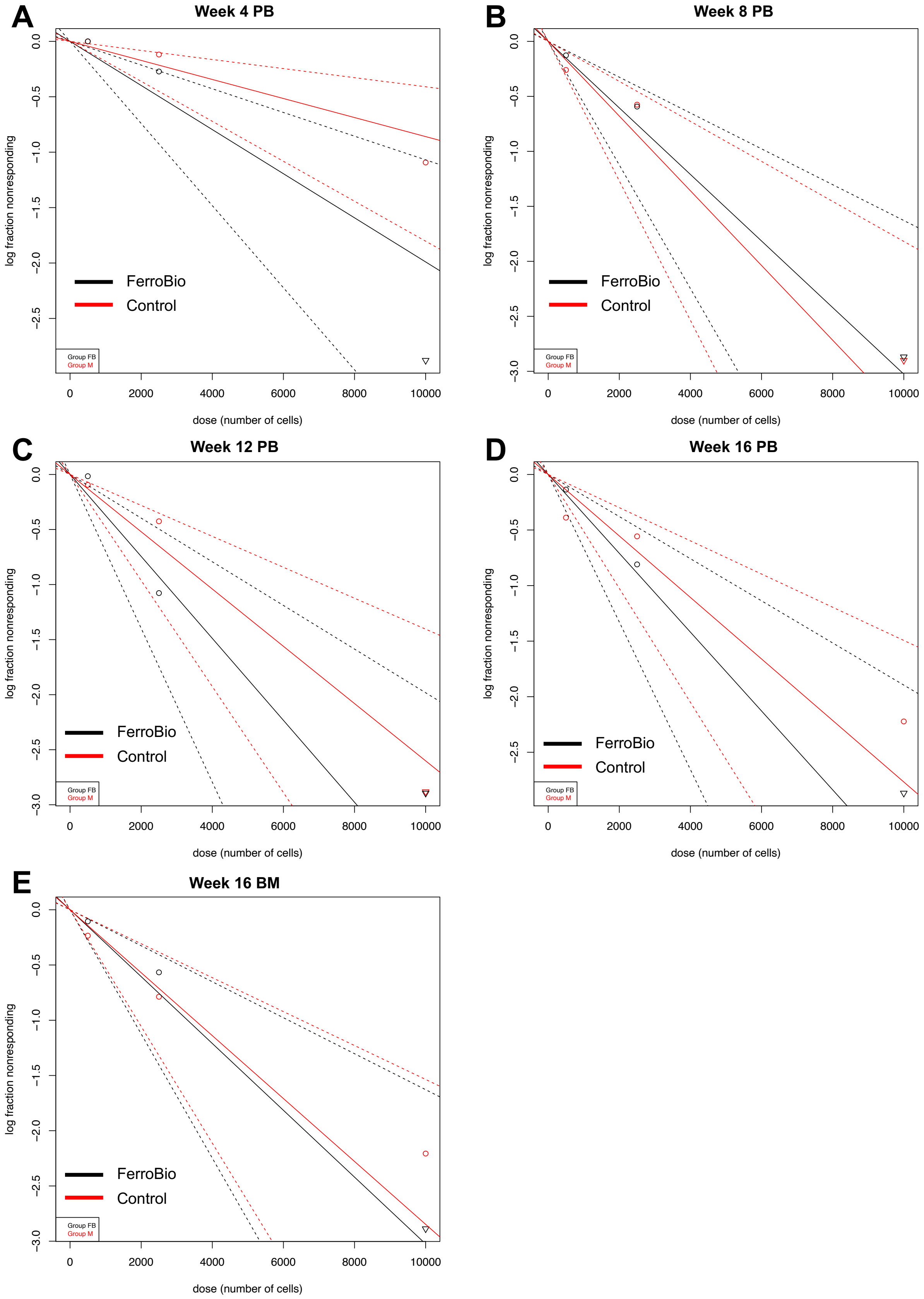

A
Week 4 PB
B
Week 8 PB
C
Week 12 PB
D
Week 16 PB
E
Week 16 BM
log fraction nonresponding
dose (number of cells)
FerroBio
Control
Group FB
Group M
0.0
−0.5
−1.0
−1.5
−2.0
−2.5
−3.0
0
2000
4000
6000
8000
10000

Supplemental Figure 4 (Related to Figure 4). A-E) Sublethally irradiated NSG mice were transplanted with three doses of CD34+ cells from FerroBio isolations or column-based control isolations. A-E) Extreme Limiting Dilution plots used to calculate SRC frequencies for the indicated time points in either peripheral blood (PB) or bone marrow (BM).

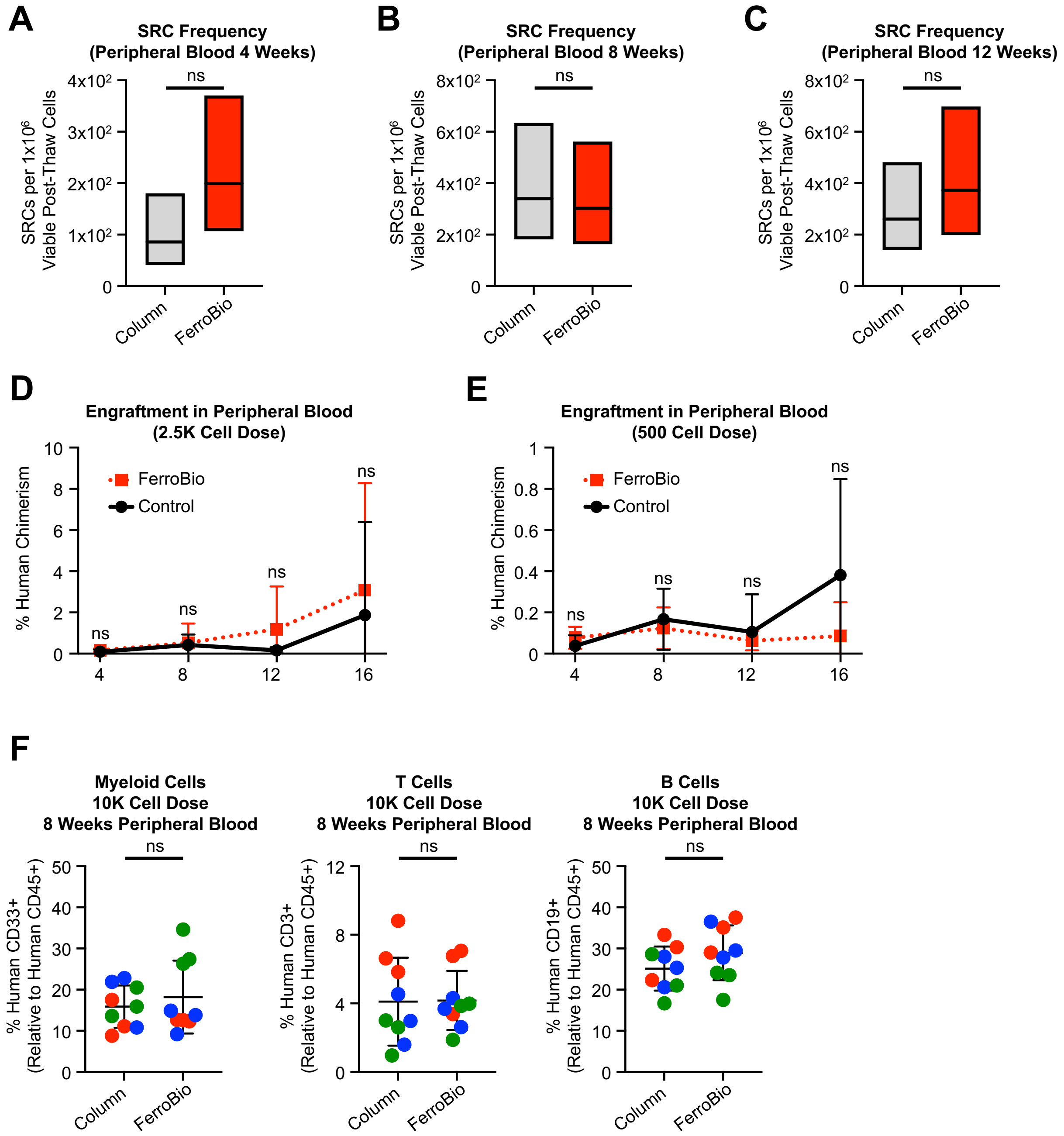

Supplemental Figure 5 (Related to Figure 5). A-F) Sublethally irradiated NSG mice were transplanted with three doses of CD34+ cells from FerroBio isolations or column-based control isolations. A-C) SCID repopulating frequencies (SRCs) in peripheral blood at the indicated number of weeks post transplantation. D) Peripheral blood engraftment over time of the medium cell dose. E) Peripheral blood engraftment over time of the lowest cell dose. F) Myeloid and lymphoid cell composition as a percentage of total human CD45+ cells in peripheral blood of mice at week 8 post transplantation. Stats: SRC frequency was compared by limdil linear modeling in R. Engraftment was compared at each individual time point comparing isolation techniques and controlling

for CBU as a random effect using linear modeling and ANOVA. ns = not significant. Each different colored point in D-F represents a different CBU.
